# Neurogenic Pulmonary Edema Presenting as a Pulmonary Entity

**DOI:** 10.7759/cureus.32002

**Published:** 2022-11-29

**Authors:** Kumaran Balasundaram, Padmavathi Parthasarathy, Gerrit Woltmann

**Affiliations:** 1 Respiratory Medicine, University Hospitals of Leicester NHS Trust, Leicester, GBR

**Keywords:** epilepsy, seizures, non-cardiogenic pulmonary edema, npe, neurogenic pulmonary edema

## Abstract

Acute dyspnea is one of the most common presentations in acute/emergency settings, and acute pulmonary edema remains a leading cause in adults resulting from either cardiogenic or non-cardiogenic etiologies. Neurogenic pulmonary edema (NPE) is one of the less common forms of non-cardiogenic pulmonary edema seen in emergency departments, neurology units, or intensive care units. It usually develops rapidly following significant neurological insult seen in patients with intracranial hemorrhage, traumatic brain injuries, and epileptic seizures. It is less commonly seen after a multitude of other sudden catastrophic neurologic insults. Here, we report a case study of a 32-year-old female with a history of epilepsy since childhood who was admitted to our respiratory admission unit on two separate occasions with acute NPE and type I respiratory failure after a witnessed tonic-clonic seizure episode. Although the clinical features of NPE and the results of investigations can mimic more common cardiorespiratory conditions, an accurate and timely diagnosis is vital for the appropriate emergency management and to improve the patient’s outcome.

## Introduction

Neurogenic pulmonary edema (NPE) is an important cause of non-cardiopulmonary acute pulmonary edema. NPE usually develops within minutes or hours following a significant insult to the central nervous system (CNS) [1]. It was first described as a complication of generalized convulsive seizure in 1908 by Shanahan, cited by Davidson et al. [2]. The published case reports and case series have identified intracerebral bleeding, traumatic brain injury, ischemic stroke, subdural hematoma, encephalitis, and epilepsy as some of the CNS triggers causing NPE [3]. Among these, cerebral hemorrhage has been reported as the most common cause of NPE, and convulsion has been the less common cause accounting for 2% of the cases [4]. NPE after convulsion is potentially life-threatening as it can lead to sudden unexpected deaths in epilepsy (SUDEP), especially in patients with frequent and recurrent seizure episodes [5]. Signs and symptoms are similar to more common forms of pulmonary edema. Significant dyspnea sometimes accompanied by hemoptysis or frothy sputum is commonly seen with physical signs of tachypnea, tachycardia, and basal crackles on auscultation. Prompt diagnosis of NPE and careful exclusion of competing cardiopulmonary etiologies contributing to the development of acute pulmonary edema are essential to improve patient outcomes. The management of NPE is mainly supportive and treating the underlying neurological problem is crucial as mortality is more commonly related to the underlying neurologic disease entity.

## Case presentation

**Figure 1:**
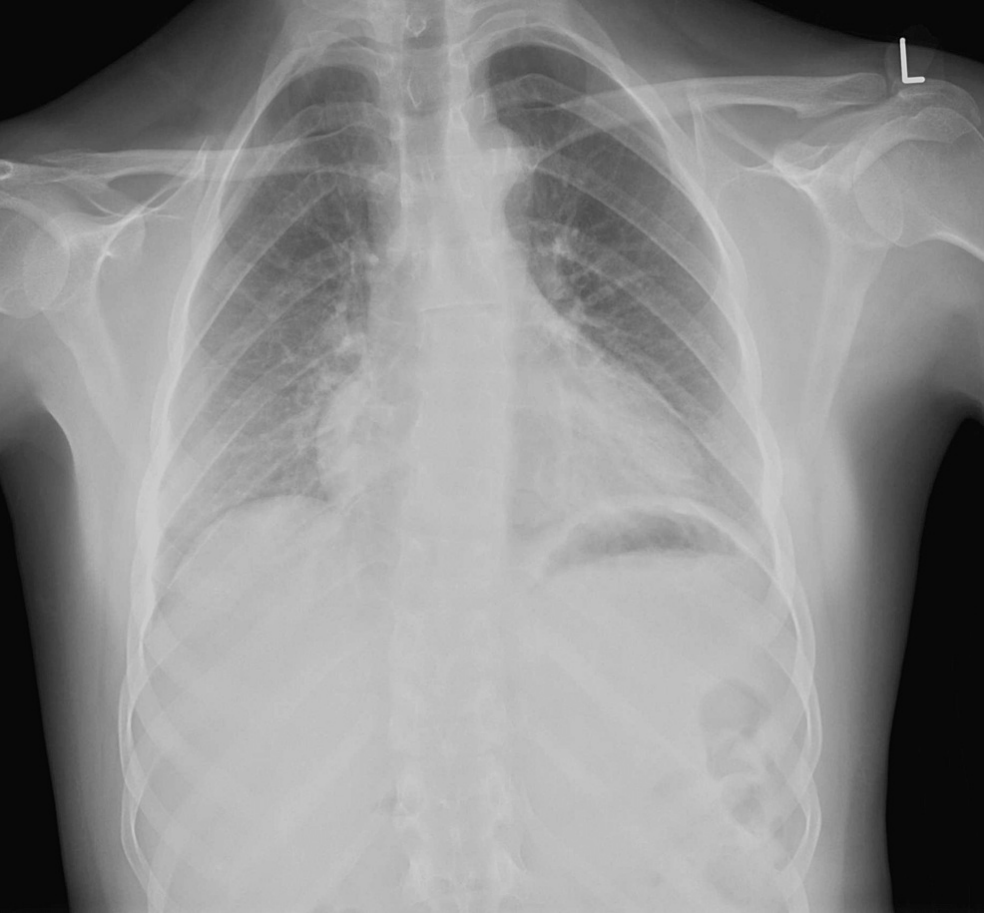
Chest X-ray before the seizure episode.

A 32-year-old female with a complex medical history of epilepsy (complex partial seizures), cerebral palsy, learning difficulty, autism, registered blind to the right eye, and right-sided hemiplegia attended the radiology department for a chest X-ray, having recently received community-based treatment for lower respiratory tract infection. Her initial chest X-ray did not show any abnormalities (Figure 1). While in the X-ray room she had a tonic-clonic seizure lasting three minutes. Post-seizure, she became severely hypoxic with saturations of 76% on room air with blood pressure (BP) of 71/46 mmHg, heart rate of 99 beats per minute, respiratory rate of 26 breaths per minute, and temperature of 37.5°C. With 15 L of oxygen via a non-rebreather mask her saturation improved to 99%.

**Figure 2:**
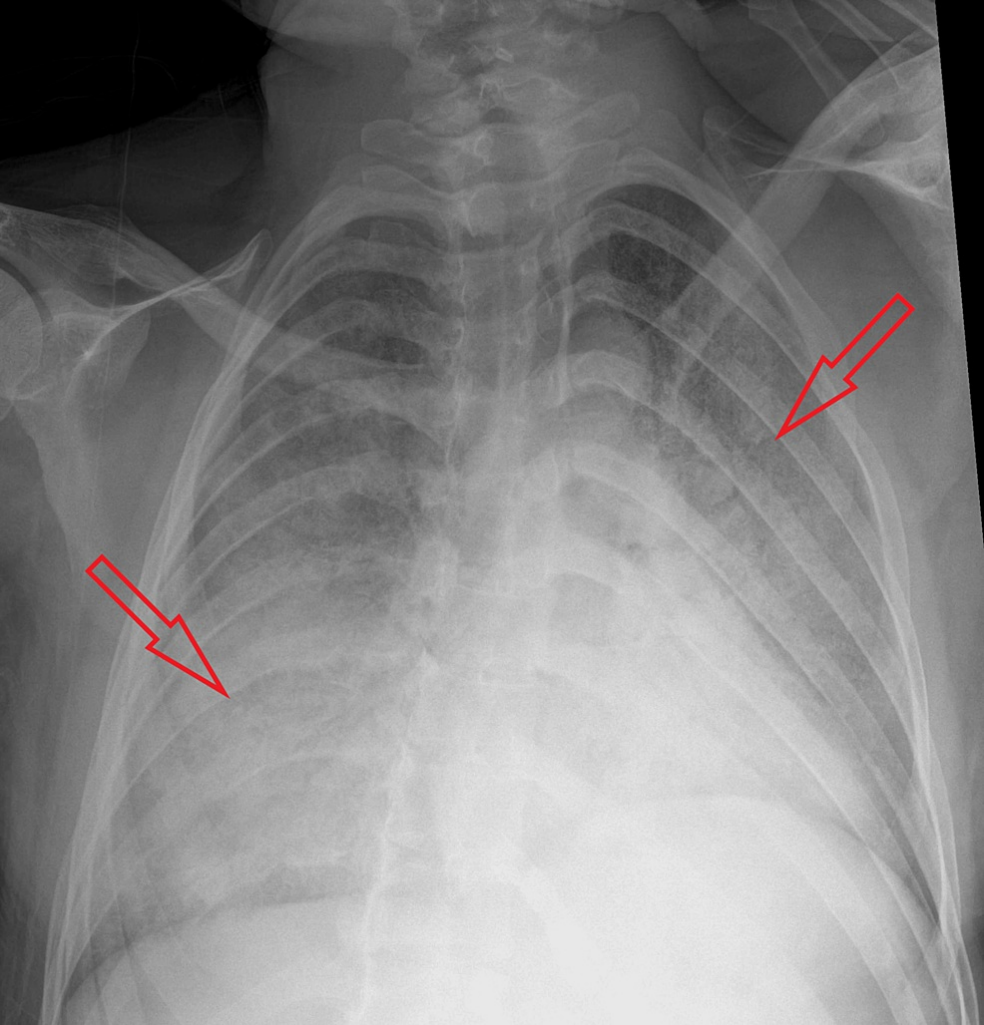
Chest X-ray after the seizure episode showing bilateral infiltrates.

She underwent a repeat chest X-ray due to her sudden deterioration which showed extensive bilateral airspace opacification (Figure 2). The case was discussed with intensive care and respiratory teams. In the clinical context, the chest X-ray changes were thought to be due to NPE. The patient was transferred to the cardiorespiratory decisions unit for further management under the care of the respiratory team. Admission blood results showed a white cell count of 27.0 × 10^9^/L, neutrophils of 24.69 × 10^9^/L, C-reactive protein of <5 mg/L, D-dimers of 1.49 µg/mL FEU, and N-terminal-pro hormone-B-type natriuretic peptide of 184 ng/L.

To cover possible aspiration pneumonia and in view of the neutrophilia, the patient was started on intravenous co-amoxiclav. Pulmonary embolism was considered a possible differential diagnosis. The patient was commenced on a therapeutic dose of low-molecular-weight heparin and a computed tomography pulmonary angiogram (CTPA) was requested. Her oxygen requirement was initially supported by high-flow oxygen via a non-rebreather mask. Due to increased oxygen demand and respiratory distress later that day, the patient was commenced temporarily on continuous positive airway pressure (CPAP) ventilation via a face mask which was poorly tolerated. Subsequently, her oxygenation failure was treated by high-flow oxygen requiring 60% FiO_2_ for the first 24 hours. After 24 hours, she started to improve with a gradual reduction in oxygen requirement.

**Figure 3:**
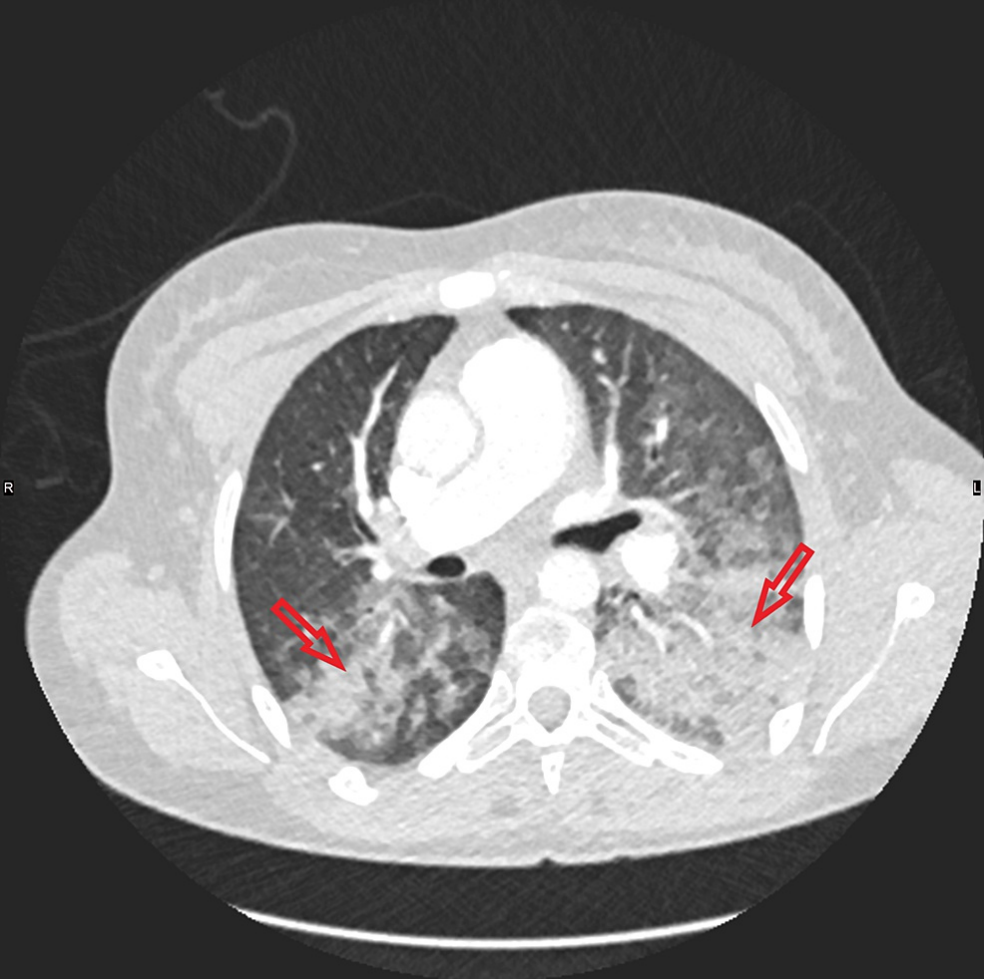
Computed tomography scan showing bilateral ground-glass opacities.

CTPA examination excluded pulmonary embolism but showed extensive bilateral opacifications within the lungs (Figure 3). The differentials reported were pulmonary edema, infective changes, eosinophilic pneumonia, and vasculitis. Based on the CT findings, the therapeutic dosing of low-molecular-weight heparin was switched to a prophylactic dose as per hospital protocol on venous thromboembolic prevention. Antibiotic treatment for aspiration pneumonia continued and prednisolone was started for hypersensitivity pneumonitis after advice from the Interstitial Lung Disease (ILD) team. Her vasculitis screening was negative. She was weaned off from supplemental oxygen on the fourth day of her admission.

**Figure 4:**
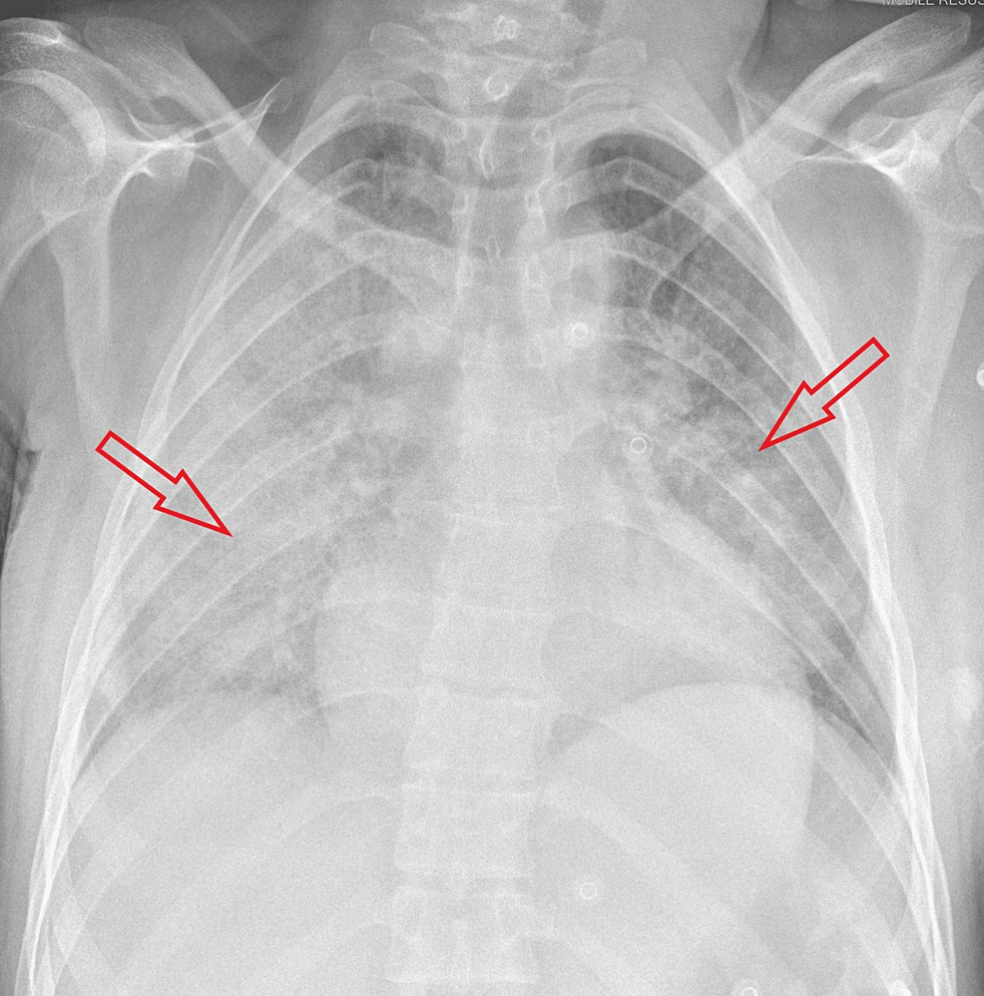
Chest X-ray from a previous admission showing bilateral infiltrates.

**Figure 5:**
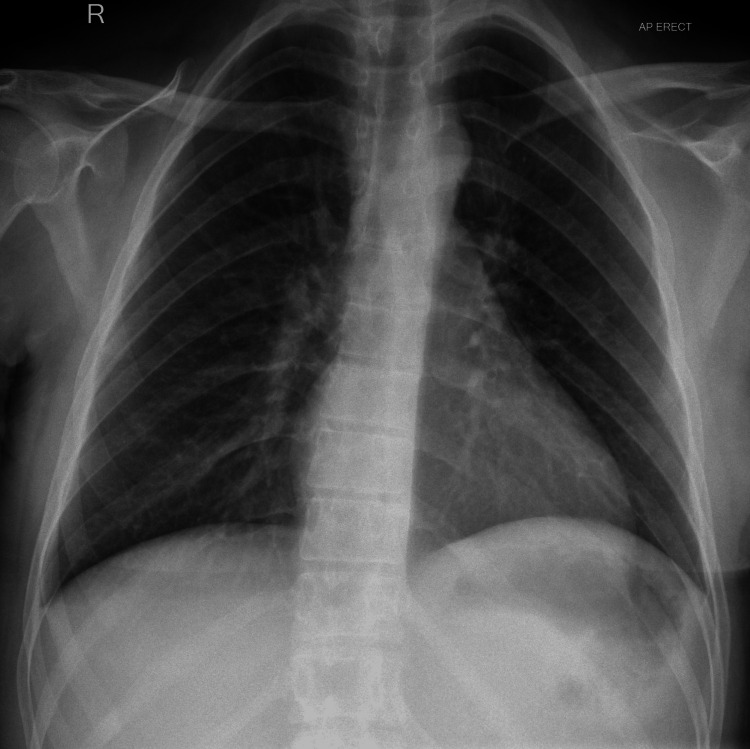
Chest X-ray before discharge during a previous admission.

From the family’s collateral history her recently increased seizure frequency became apparent and two other recent admissions with very high levels of oxygen requirement, one needing intensive care admission and mechanical ventilation. During those admissions, she was treated for sepsis and atypical infection consecutively as the chest X-ray showed bilateral infiltrates (Figure 4). However, the chest X-ray was completely clear within a few days of treatment (Figure 5). NPE was overlooked as a differential diagnosis and there were missed opportunities to optimize her epilepsy management.

**Figure 6:**
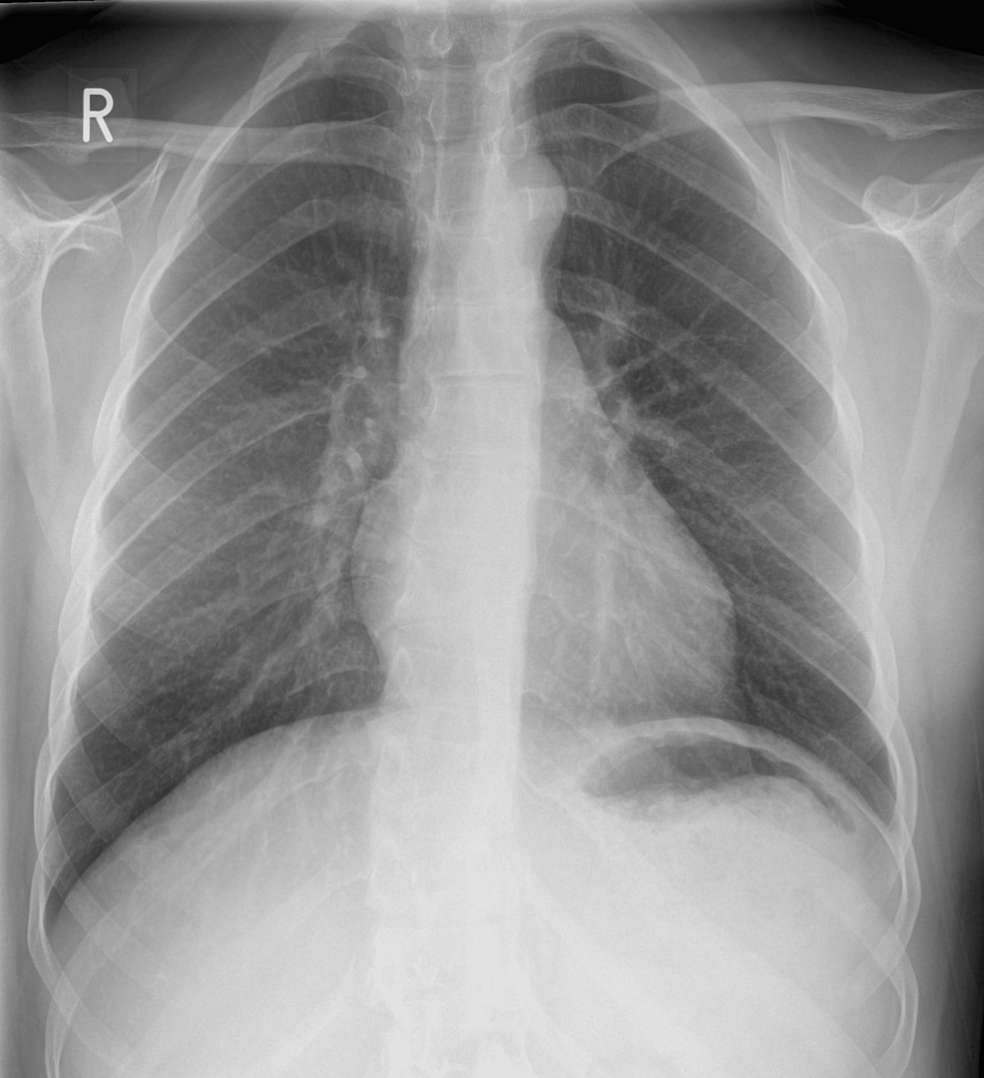
Chest X-ray before discharge showing clear lung fields (current admission).

During the current admission, her ongoing seizure management was discussed with the Neurology team. They assisted to optimize her epilepsy and arranged for outpatient investigations and follow-ups. The patient was discharged after seven days with a clear chest X-ray (Figure 6).

## Discussion

NPE is a well-documented, life-threatening, early complication of CNS insult. It typically develops within minutes to hours after such events and is associated with raised intracranial pressure [3]. It has been observed most commonly in patients with subarachnoid hemorrhage or other causes of intracerebral hemorrhage, traumatic brain injury, ischemic stroke, encephalitis, and epilepsy [3]. Common clinical manifestations include tachypnea, tachycardia, crackles on physical examination, minimal leucocytosis, and subsequent progression to hypoxemic respiratory failure [6]. The chest radiograph frequently shows bilateral widespread infiltrates without cardiomegaly [7]. Delayed presentations of pulmonary edema 12 to 72 hours following a CNS event are also recognized [8].

Pathophysiologically, NPE represents fluid accumulation within the interstitium and air spaces of the lungs often developing rapidly after acute CNS insults. It classically presents as a diagnosis of exclusion due to the complexity of its pathophysiological mechanisms involving hemodynamic, CNS, and systemic inflammatory changes [9]. However, a differential diagnosis of NPE should be included early when a patient presents with sudden onset of dyspnea and new oxygen requirement [10] after sudden neurologic insult. In patients with epilepsy, NPE is more common after status epilepticus [11]. Other differential diagnoses include hypersensitivity pneumonitis, aspiration pneumonia, atelectasis, and cardiogenic pulmonary edema [3]. NPE is considered to be one of the major causes of SUDEPz, and one study found 52 out of 72 autopsies of SUDEP patients had marked pulmonary edema [10,11].

There is an ongoing debate regarding how neurological insult results in pulmonary edema. There are a few theories explaining the phenomena of pulmonary edema after an epileptic seizure episode. A neuro-hemodynamic mechanism theory known as blast theory is one of the earliest theories and suggests intense pulmonary and systemic vasoconstriction as a response to the sympathetic surge, leading to increased capillary hydrostatic pressure and permeability (starling) in the lungs [12]. These changes lead to pulmonary capillary endothelial damage and leakage of protein-rich fluid into the alveoli [13]. Another theory suggests that during a generalized seizure, a sudden rise in intracranial pressure occurs which increases the sympathetic activation resulting in NPE. In adults, NPE is more frequently observed with prolonged and frequent seizure activities [14]. According to another proposed mechanism, the cytokines (pro-inflammatory molecules) are released within the brain following an insult, interrupt the blood-brain barrier, and reach the lung via systemic circulation. These cytokines damage the endothelial cells leading to increased capillary permeability and resulting in pulmonary edema [9]. The excessive negative intrathoracic pressure caused by the inspiratory effort against a closed glottis and prolonged increase in intrathoracic negative pressure gradients can lead to a fluid shift from capillaries to the lung parenchyma resulting in pulmonary edema [15]. Management of NPE should focus on the treatment of the underlying neurological issue and ventilation support.

NPE should be suspected in a patient who suffered a neurological insult after excluding other cardiopulmonary causes for dyspnea. Further, the diagnosis of NPE is warranted when there is radiographic evidence of bilateral pulmonary infiltrates and the presence of hypoxemia.

Our patient rapidly deteriorated following a witnessed episode of tonic-clonic seizure without reported vomiting. Our initial differential diagnoses were aspiration pneumonia and pulmonary embolism with other differentials later considered after CT scanning including eosinophilic pneumonia and hypersensitivity pneumonitis. While she was treated with antibiotics for suspected aspiration pneumonia due to high white cell counts, the elevated inflammatory markers could have been due to the release of pro-inflammatory cytokines during the neurological insult. She was also transiently treated with prednisolone as recommended by the ILD team but NPE was the most likely diagnosis.

## Conclusions

NPE is an uncommon presentation in respiratory admission units. It should be considered as a differential diagnosis in patients who present with dyspnea, tachycardia, and hypoxia following sudden cerebral insults including seizures after excluding other cardiorespiratory diagnoses. NPE in seizure patients is directly related to the frequency and duration of seizure episodes and is one of the causes of SUDEP in this patient group. NPE secondary to seizure requires supportive care and better seizure control.
